# Decentralized Platoon Join-in-Middle Protocol Considering Communication Delay for Connected and Automated Vehicle

**DOI:** 10.3390/s21217126

**Published:** 2021-10-27

**Authors:** Geonil Lee, Jae-il Jung

**Affiliations:** 1 Department of Electronics and Computer Engineering, Hanyang University, 17 Haengdang-dong, Seongdong-gu, Seoul 133-791, Korea; lgi1992@hanyang.ac.kr; 2 Department of Electronic Engineering, Hanyang University, 17 Haengdang-dong, Seongdong-gu, Seoul 133-791, Korea

**Keywords:** connected and automated vehicle, platooning system, vehicle-to-vehicle communication, platoon management, platoon join-in-middle protocol, trajectory planning, variable time headway

## Abstract

Cooperative driving is an essential component of intelligent transport systems (ITSs). It promises greater safety, reduced accidents, efficient traffic flow, and fuel consumption reduction. Vehicle platooning is a representative service model for ITS. The principal sub-systems of platooning systems for connected and automated vehicles (CAVs) are cooperative adaptive cruise control (CACC) systems and platoon management systems. Based on vehicle state information received through vehicle-to-vehicle (V2V) communication, the CACC system allows platoon vehicles to maintain a narrower safety distance. In addition, the platoon management system using V2V communications allows vehicles to perform platoon maneuvers reliably and accurately. In this paper, we propose a CACC system with a variable time headway and a decentralized platoon join-in-middle maneuver protocol with a trajectory planning system considering the V2V communication delay for CAVs. The platoon join-in-middle maneuver is a challenging research subject as the research must consider the requirement of a more precise management protocol and lateral control for platoon safety and string stability. These CACC systems and protocols are implemented on a simulator for a connected and automated vehicle system, PreScan, and we validated our approach using a realistic control system and V2V communication system provided by PreScan.

## 1. Introduction

In the research field of autonomous vehicles, cooperative driving technology using communication between vehicles has attracted considerable attention. Through the adaptation and development of cooperative driving technologies in the perception, decision-making, and control layers of autonomous vehicles, intelligent transport systems (ITSs) improve vehicle safety, traffic efficiency, and fuel efficiency, which is called Cooperative-ITS (C-ITS). The typical system of C-ITS is platooning, which allows several grouped vehicles to maintain a narrow distance between them and drive at the same speed. The narrow gap between the platooned vehicles leads to a decrease in air resistance, which reduces the fuel consumption of the platoon vehicles. In addition, the consensus-based platoon maneuver between platoon vehicles improves traffic flow and safety [1,2]. In general, most research in platooning systems focuses on string stability through the control system and platoon management through the vehicle-to-everything (V2X) communication system.

The core of the control system for platoons is the adaptive cruise control (ACC) system. The ACC system calculates the distance and speed from the preceding vehicle (PV) using on-board sensors, such as radar sensors, and automatically adjusts the vehicle speed to maintain a safe distance from the PV. Recently, the cooperative ACC (CACC) system has become an important research topic as it enables the sharing of kinematic information between vehicles through vehicle-to-vehicle (V2V) communication. The kinematic information of PV allows the vehicle to perceive the change in the motion of PV more accurately and rapidly, thereby allowing the host vehicle (HV) to maintain a narrow distance with safety guarantees [3,4]. 

The platoon management system is key to properly supporting platoon maneuvers of platoon vehicles. The system enables the performance of platoon maneuvers such as generation, merging, splitting, joining, and leaving of the platoon through dedicated protocols and platoon information (platoon ID, platoon size, platoon member, platoon speed, etc.) management. Based on the exchange of vehicle state information and maneuver protocol messages through V2X communication, the platoon management system supports a platooning system safely and efficiently [5].

The platooning system is designed in accordance with the V2X communication topology [6,7,8]. Typically, in a centralized communication topology, the leader vehicle that creates a platoon and leads the vehicles in the platoon determines all actions, and the member vehicles communicate directly with the leader to receive the platoon information and decisions. Here, the leader vehicle gathers information from the member vehicles periodically, determines the appropriate behavior of the member vehicles, and relays the decisions. However, owing to the characteristics of wireless communication, the low packet delivery rate (PDR) caused by the long communication distance from the leader results in high retransmission delays and reduces string stability. In addition, because the leader vehicle determines all actions, computation overheads of the leader vehicle and limited execution of the simultaneous maneuver occur, which can lead to a high communication delay that can affect the platoon maneuver [9]. Therefore, we designed a platooning system based on a decentralized communication topology where each member vehicle communicates directly with the PV and the following vehicle (FV). It enables member vehicles to have rapid perception and decision based on low communication delays and low packet error rate and to drive while maintaining a narrow intra-platoon distance. 

Many studies have been conducted on platooning systems using decentralized communication topology [10,11,12]. However, most studies do not consider communication delays or packet losses. Although the decentralized approach resolves the high communication delay problem caused by long communication distances from the leader, network delays can still occur depending on channel congestion and the surrounding environment. Therefore, V2X communication delays and packet loss must be considered for safe platooning [13,14]. Hu et al. determined the actual communication delay through field tests and modeled the minimum safe distance according to the communication delay in acceleration, cruising, and deceleration states [15].

Many studies have investigated the platoon join maneuver, which occurs at the tail or head of a platoon. However, the maneuver is uncomplicated because it only considers longitudinal control [5,16,17,18]. Research on advanced platoon management protocols using lateral control is required to ensure efficient traffic flow. In the cases of highway on-ramp or lane reduction due to an obstacle, a platoon management protocol is required to support joining in the middle of the platoon [19]. Hidalgo et al. proposed a cooperative platoon merging maneuver based on the CACC system and trajectory planning [20].

In this study, we estimate the communication delay utilizing the cumulative communication delay strategy and apply the delay to a variable time headway for the CACC system, time-out interval, and trajectory planning for the platoon maneuver protocol. In particular, we implemented a join-in-middle maneuver protocol among many platoon maneuvers. The proposed protocol includes (1) a direct and reliable consensus between maneuvering vehicles using a decentralized communication topology, (2) precise trajectory planning using cumulative communication delay, and (3) lane changing through lateral control. 

The developed platooning system for connected and automated vehicles (CAVs), including the CACC system and maneuver protocol, was validated on the autonomous driving simulator PreScan [21]. The simulation results show that the HV joins reliably in the middle of the platoon, maintaining string stability through a sophisticated control system and communication system supported by PreScan.

The remainder of this paper is organized as follows. The background of the decentralized platoon join-in-middle maneuver protocol with a variable time headway is presented in Section 2. The details of the proposed platooning system are presented in Section 3. Section 4 describes the simulation environment and evaluation results of the platoon join-in-middle maneuvering. Finally, concluding remarks are presented in Section 5.

## 2. Background: Motivation and Related Work

**Figure 1:**
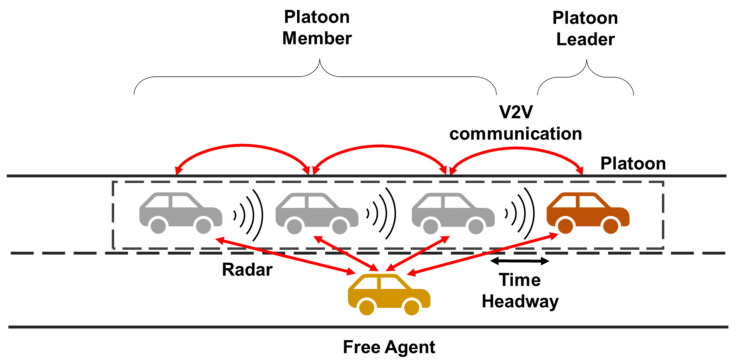
Decentralized platooning system of connected and automated vehicles.

In our study, we used a decentralized communication topology to design a platooning system for CAVs as shown in Figure 1. The platoon member vehicle uses the vehicle state information received from the PV for the CACC system. The free agent also periodically communicates with nearby vehicles before participating in the platoon. After deciding to participate in the platoon, the free agent selects PV and FV based on its current position and performs a join-in-middle maneuver by exchanging protocol messages with those vehicles. In this section, along with a brief background, we explain the distinct features of our approach.

### 2.1. Decentralized Platooning System

The flow topology of information over V2X communication has a significant impact on the design of platooning systems for CAVs. The source of information used in the control and platoon management systems varies depending on the topology, which affects the inter-vehicle distance, reaction time, management complexity, and maneuverability. Two approaches typically exist: the centralized approach, where the leader takes the platoon management and decision, and the decentralized approach, where each platoon member vehicle manages platoon information and determines its behavior. 

There are many existing centralized platoon systems [22,23,24]. However, owing to the characteristics of wireless communication, the PDR decreases as the distance between the leader and member vehicle increases, which causes significant communication delays owing to packet retransmission. In such a situation where communication between the leader and the member vehicle is unstable, platooning according to the information from the leader is not possible, and the safety and efficiency of the platoon are significantly reduced. 

To address this problem, a decentralized platoon maintenance approach is being investigated. Won et al. [17] suggested a method to address the degradation of leader–member communication performance in long platoons by designating a member vehicle that has a lower communication PDR with the leader as a virtual leader. However, platoon management is still conducted on vehicles that act as leaders, and frequent selection of leaders in poor communication conditions can further exacerbate channel conditions. Fida et al. proposed an improved multiple management protocol that supports multiple maneuvers simultaneously through a decentralized approach [10]. The protocol handles multiple join and leave maneuvers that occur simultaneously through V2I communication with the maneuvering vehicle and road side unit (RSU). However, because all information is transmitted to the RSU and all maneuvers are performed through the RSU, the processing time of the maneuver can be extended. In addition, the surrounding dynamic environment of the maneuvering vehicle may not be reflected in real time. Therefore, we have designed a decentralized platooning system that allows each vehicle to independently manage the platoon information based on bidirectional communication with the PV and FV and performs platoon maneuvers reliably by exchanging protocol messages between maneuver-related vehicles.

### 2.2. Variable Time Headway for CACC System

The spacing strategy, one of the key parameters of the CACC system, determines the distance from the PV and enables the controller to calculate the target speed. The spacing strategy should be reasonably designed because it directly affects the safety and efficiency of the platooning system. The spacing strategies are divided into two types: the constant spacing strategy, which maintains the inter-vehicle distance constantly, and the variable spacing (VS) strategy, which modifies the inter-vehicle distance by considering the driving environment [25]. 

The VS strategy, which is primarily used in CACC systems, determines the intra-platoon distance based on the time headway. Because the intra-platoon distance changes depending on the vehicle speed, stable platooning is possible. The VS strategy that sets the time headway to a certain value is called the constant-time headway (CTH) strategy, which is used in many platooning system studies [26,27]. However, to design a more realistic platooning system, complex variables such as traffic flow, network delay, and CAV processing time must be considered, which is called the variable time headway (VTH) strategy. Wang and Rajamani proposed an improved VTH strategy considering traffic density [28]. Yanakiev and Kanellakopoulos proposed a VTH strategy considering the relative speed between the HV and PV [29]. 

In CACC systems utilizing the speed and acceleration values of PV received via V2V communication, communication delay can also have a significant impact on the decision of the time headway [30]. If a communication channel is congested and a high communication delay occurs, the HV should have a variable time headway depending on the condition of the channel because it cannot be informed in time. Therefore, we applied a variable time headway that considers the estimated communication delay with the PV in the CACC system.

### 2.3. Platoon Join-in-Middle Maneuver Protocol

The characteristic of a platooning system for CAVs is that the exchange of maneuver protocol messages allows CAVs to perform platoon maneuvers, such as join, leave, merge, and split, efficiently and safely. In particular, the maneuver protocol for joining the platoon in another lane supports the safe lane-changing operation of the joining vehicle (JV). In this case, the JV can attempt to change the lane after moving to the head or tail of the platoon such that it does not affect the platoon member vehicles. However, if it is difficult to reach to the head or tail of the platoon owing to the traffic flow of the current lane, the JV should attempt to join in the middle of the platoon. 

The join-in-middle protocol is more challenging than other maneuvers because it requires the creation of a gap and lane changing based on precise time calculation and consensus between the JV and FV. Table 1 summarizes the recent studies on the join-in-middle maneuver protocol. Liu et al. proposed a join-in-middle maneuver protocol using merge, split, and lane change actions gradually [31]. However, a centralized topology can cause a time delay in opening the gap and changing lane, and various interferences can occur during the delay. Segata et al. designed a protocol for a join-in-middle maneuver considering various interferences [32]. However, because the leader vehicle manages the maneuver, it cannot support multiple maneuvers simultaneously. For example, while one vehicle joins the platoon, the other vehicle cannot perform a join or leave maneuver. Renzler et al. proposed an efficient maneuver protocol in a dynamic environment using a decentralized topology [13]. However, safe trajectory planning for lane changing is as important as the design of the protocol in the CAV platoon join-in-middle maneuver management system. Therefore, in this paper, we design the join-in-middle maneuver protocol for the CAV, which includes the efficient opening gap strategy of FV, safe lane-changing trajectory planning of JV, considering the dynamic surrounding environment and communication delay. 

## 3. Decentralized Platoon Join-in-Middle Protocol with Variable Time Headway

### 3.1. Architecture of a Platooning System for Connected and Automated Vehicle

The structure of the CAV comprises three layers: perception, decision-making, and control layers [33]. In the perception layer, sensors (Radar, Lidar, GPS, Camera, etc.) perceive the surrounding environment and perform positioning and map construction. The information is passed on to the decision-making layer and used for global planning, behavioral planning, and local planning. When a trajectory is created through behavioral planning and local planning, the CAV is driven along the path through the controller. 

**Figure 2:**
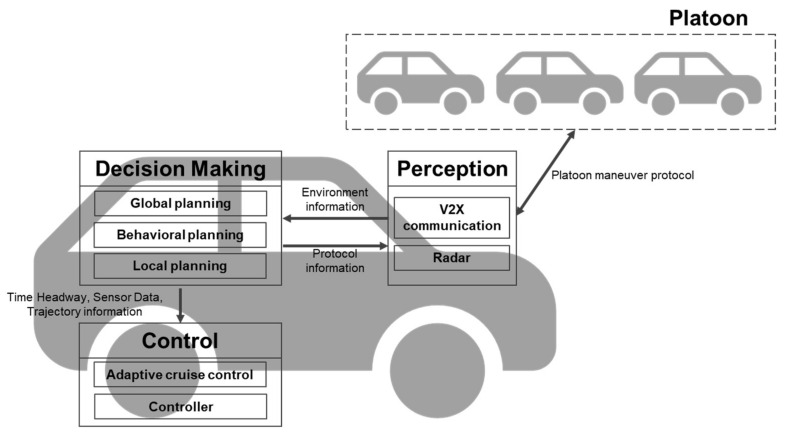
An architecture of a platooning system for connected and automated vehicles.

Figure 2 shows the architecture of a platooning system for CAVs. We assume that the behavioral planning system determines the join-in-middle maneuver and selects FV and PV, which are the destination of the protocol message, based on the current position. The scope of our study is the exchange of join-in-middle control protocol messages over V2V communication, variable time headway calculation and trajectory generation in local planning, and control including the CACC system.

### 3.2. Estimation of Communication Delay

CAVs broadcast state information periodically through V2V communication. This information is contained in the basic safety message (BSM) format, which complies with the SAE J2735 standard [34]. In our plating system, the BSM is used to calculate the communication delays. The platoon member vehicle receives a BSM from its PV, records a communication delay on the platoon table with the state information of the PV, and utilizes the information to calculate the variable time headway and target speed for the CACC. Non-platooned vehicles record the V2V communication delay and location information of all surrounding CAVs in the neighbor table, as in our previous study, and utilize the neighbor table to select the target vehicle and create the trajectory required to perform the platoon join-in-middle maneuver [33]. The communication delay was calculated as a weighted average.

(1) $$updated\_t_{w,i}\leftarrow \left(1-\;\alpha \right)\times t_{w,i}+\alpha \times t_{r,i},$$

where i is the CAV identification, $t_{w,i}$ is the weighted average communication delay with CAV i, $t_{r,i}$ is the latest end-to-end delay with CAV i. Deviations $dev_{w,i}$ are also calculated to deal with the variability of the communication delay.

(2) $$updated\_dev_{w,i}\leftarrow \left(1-\;\beta \right)\times dev_{w,i}+\beta \times \left|t_{r,i}-t_{w,i}\right|,\;$$

In this study, we further considered time-out events to cope with the failure of protocol behavior owing to packet loss and high communication delay that can occur in V2V communication. To set the time-out interval, we modified the time-out interval formula used in computer networks [35].

(3) $$t_{time\;out}=2\times \underset{i}{\max}t_{w,i}+8\times {\mathrm{dev}}_{w,\mathrm{argmax}t_{w,i}},\;$$

### 3.3. Variable Time Headway for CACC System

The platooned vehicle recognizes kinematic information such as location, speed, acceleration, and deceleration of the vehicle ahead through V2V communication in real time, calculates the safe distance based on it, and transmits it to the CACC controller. The perception time is determined by the communication delay with the PV and has a changing value depending on the channel status. In our system, an estimated communication delay with the PV was used in the calculation of the time headway for safety distance, which improved the stability of the system.

(4) $$t_{headway}=t_{default}+t_{w,PV}+{dev}_{w,PV},$$

where $t_{headway}$ is the time headway considering the communication delay, and $t_{default}$ is the default time headway of the CACC system. However, if the estimated communication delay in the platoon table is not updated during two consecutive periods owing to packet loss, the system determines that there is a problem with V2V communication and uses the time headway of the ACC system, which utilizes the radar sensor instead of the CACC time headway.

### 3.4. Decentralized Platoon Join-in-Middle Protocol

**Figure 3:**
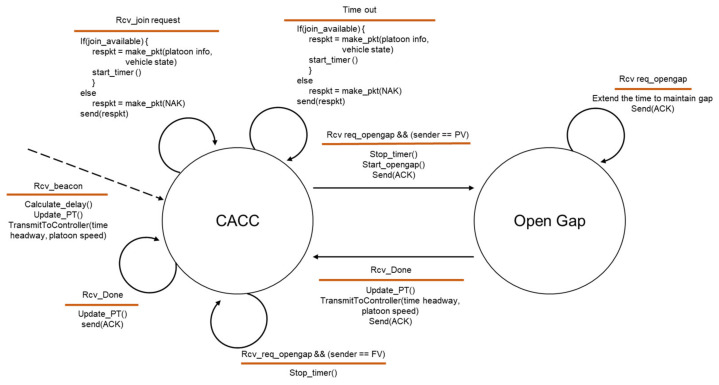
State machine of platoon member vehicle.

**Figure 4:**
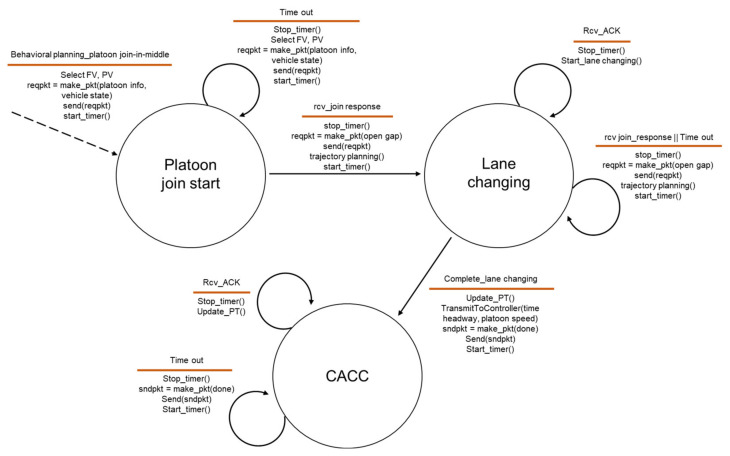
State machine of joining vehicle.

After the perception of the platoon driving in the next lane and the determination of the join-in-middle maneuver in the behavioral planning, the HV selects PV and FV based on their current position and performs a join-in-middle maneuver by exchanging protocol messages with them. Figure 3 and Figure 4 show the final state machine of the join-in-middle maneuver protocol. Figure 3 shows the state machine of the member vehicle, and Figure 4 shows the state machine of the JV.

Two states exist for the member vehicle: CACC and open gap. The member vehicle calculates the communication delay based on the BSM received from the PV, updates the platoon table, and drives while maintaining a safe distance. When the member vehicle receives a join request message from the JV, the member vehicle provides platoon information and vehicle state information to the JV through a join response message if the joining is allowed. The member vehicle allows the JV to join if the member vehicle is driving at a constant speed without any other maneuvering in progress. When an open gap request message is received in response to a join response message, the FV starts decelerating to create a gap and transitions to an open gap state. If a duplicate open-gap request message is received in the open-gap state, the FV extends the time to maintain the gap. When a lane-changing-done message is received from the JV, the FV transitions to the CACC state after updating the platoon table. Both PV and FV finish the join-in-middle maneuver by sending an acknowledgment (ACK) message for the lane-changing-done message from JV.

Three states for joining vehicles exist: the platoon join start, lane changing, and CACC. When the JV determines the platoon join-in-middle maneuver, it selects PV and FV and transmits a join request message to them. When the JV receives a response message from the PV and FV, it sends an open-gap request message and plans a trajectory for the join-in-middle based on the PV and FV state information and platoon information. After receiving an ACK message from the FV in the lane-changing state, JV performs lane changing along the expected trajectory. When the JV joins the platoon via lane changing, it becomes a CACC state and transmits a lane-changing-done message to the PV and FV, confirming that it has joined the platoon. With the receipt of ACK messages for the lane- changing-done messages, JV finishes the join-in-middle maneuver and becomes a member vehicle.

We designed the join-in-middle maneuver to be stable through a time-out event and ACK message exchange dealing with high communication delay or packet loss during protocol progress.

### 3.5. Local Planning for Platoon Join-in-Middle Protocol

**Figure 5:**
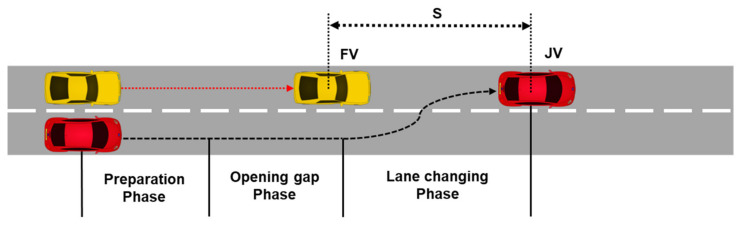
Construction of the expected trajectory.

We design the join-in-middle trajectory in three phases: preparation, opening gap, and lane-changing phases. Figure 5 shows the expected trajectory for the join-in-middle maneuver generated by the local planning.

First, in the preparation phase, JV calculates the time of each of the three phases and determines the position, speed, and steering value based on the time required to generate the trajectory. The preparation phase begins the moment the JV receives a join response message from the FV. This is because the deceleration, acceleration, and speed information contained in the join response message of the FV are required to calculate the delay of the opening gap phase. It also provides flexibility in dealing with the modification of the trajectory owing to the retransmission of the message. Therefore, the preparation delay is defined as the sum of the communication delay, delay variance, and processing delay of JV and FV.

(5) $$t_{prepare}=t_{w,FV}+dev_{w,FV}+t_{processing,JV}+t_{processing,FV},$$

where $t_{w,FV}$ is the communication delay with the FV on the neighbor table and $dev_{w,FV}$ is the deviation of the communication delay with FV, $t_{processing,JV}$ is the processing time at which JV generates the entire trajectory, and $t_{processing,FV}$ is the time at which FV calculates the size of the gap and determines the deceleration based on the platoon information. During this phase, it is assumed that all vehicles are driven at the same speed.

Second, in the opening gap phase, the FV decelerates to open the gap such that the JV can enter the platoon. The strategy for calculating the delay in the opening gap phase is as follows: The JV maintains the longitudinal speed at the platoon speed and changes lanes. The opening gap phase of the FV comprises time $t_{1}$, at which the deceleration to open the gap of the FV is finished, and time $t_{2}$, at which the FV reaches the platoon speed through acceleration. At the end of the acceleration, the FV must maintain an intra-platoon distance with the JV. The opening gap delay of JV is time $t_{1}$ at which the FV has reached the minimum speed, and the JV starts the lane changing at $t_{1}$. Consequently, the trajectory is designed to enable the JV to safely complete the join-in-middle maneuver in the shortest time without disturbing while the gap is open. 

**Figure 6:**
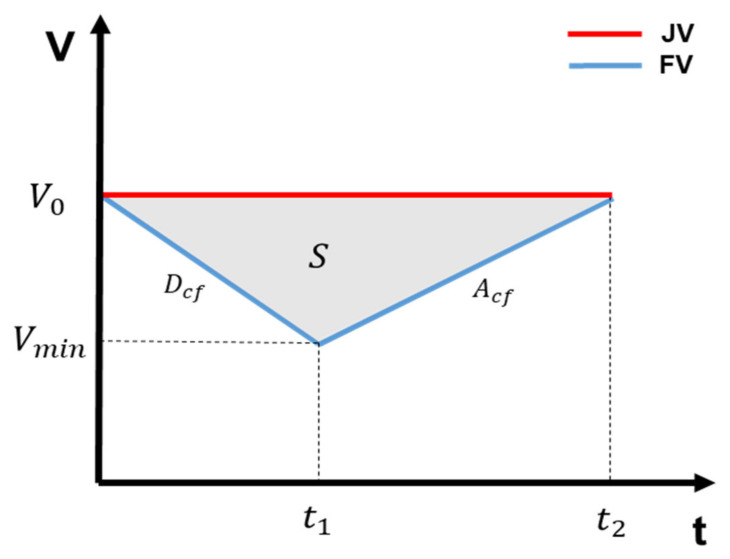
Speed changes of the JV and FV in the opening gap phase.

Figure 6 shows the speed changes of the JV and FV in the opening gap phase for the calculation of the opening gap delay.

(6) $$S=\frac{t_{2}\times \left(V_{0}-V_{min}\right)}{2},\;$$

where S is the distance difference between the JV and FV when the joining is complete, $V_{0}$ is the platoon speed, and $V_{min}$ is the minimum speed of the FV during the opening gap phase.

(7) $$S=t_{headway}\times V_{0}+{\;D}_{standstill}+\frac{L_{JV}+L_{FV}}{2},\;$$

When the joining is complete, S is the intra-platoon distance. $D_{standstill}$ is the minimum distance between the platoon vehicles. $L_{JV}$ and $L_{FV}$ are the lengths of the JV and FV, respectively.

(8) $$t_{2}=t_{1}\times \left(\frac{A_{cf}-D_{cf}}{A_{cf}}\right),\;$$

(9) $$V_{min}=V_{0}+D_{cf}\times t_{1},\;$$

where $A_{cf}$ represents the comfort acceleration of the FV and $D_{cf}$ denotes the comfort deceleration of the FV. The opening gap delay of the JV is $t_{1}$, given by the formula below.

(10) $$t_{opengap}=t_{1}=\sqrt{\frac{2A_{cf}\times \;S}{D_{cf}\left(D_{cf}-A_{cf}\right)}},$$

Third, in the lane-changing phase, the lane-changing path of the JV is defined. We used the lane-changing path applied in our previous lane-change protocol study [33]. The ramp sinusoidal function was used for the ideal lateral movement.

(11) $$y_{p}\left(t\right)=\frac{y_{e}}{x_{e}}V_{0}t-\frac{a_{y}x_{e}^{2}}{4\pi V_{0}^{2}}\sin\frac{2\pi V_{0}t}{x_{e}}(\;t_{lanechange}<t<t_{end}\;),$$

(12) $$x_{p}(t)=V_{0}t(\;t_{lanechange}<t<t_{end}\;),$$

where $x_{p}$(t) is the longitudinal displacement of the JV, $y_{p}$(t) is the lateral displacement of the JV, $x_{e}$ is the longitudinal distance of the lane change, $y_{e}$ is the end of the lateral displacement (equivalent to the lane width)$,\;t_{end}$ is the time when the vehicle joins the platoon, and $a_{y}$ is the lateral acceleration. The normal lateral acceleration equation was used for the comfort acceleration.

(13) $$a_{y}\left(t\right)=(0.025V-\left(\frac{V_{0}}{44}\right))g,$$

and the total longitudinal distance proposed by Limpert is utilized.

(14) $$x_{e}=C_{x}V_{0}\sqrt{\frac{y_{e}}{a_{y}}},$$

### 3.6. Controller

In our platooning system, CAV uses PID control for longitude control. The ACC system of the platoon member vehicle sets the speed of the PV received via V2V communication as the target speed and controls the speed using the PID error, which is the difference between the current and target speeds. In addition, standstill distance and variable time headway are provided as inputs to the ACC system; thus, if the inter-vehicle distance recognized through the radar is reduced below the safety distance, CAV decelerates to maintain a safe distance considering the relative speed of the PV. If the inter-vehicle distance is longer than the safety distance, the ACC system narrows the distance by setting a higher target speed. All maneuvers are controlled with the appropriate margin such that the speed fluctuations of the vehicle are not large.

Pure pursuit control was used as a lateral control method for lane changing. The pure pursuit algorithm calculates the steering angle using a look-a-head point to follow the expected trajectory, and the steering angle is passed to the vehicle dynamics model.

## 4. Evaluation

### 4.1. Experimental Setup

We used Simcenter PreScan to validate the proposed platooning system. PreScan provides a variety of sensor systems, including V2V communication, vehicle dynamics, and control functions, to simulate CAV systems. In particular, it provides sophisticated vehicle dynamics and control functions compared to traffic simulators and network simulators; thus, the influence of platoon maneuvers on string stability can be evaluated more realistically. The 2D simple dynamics model was used as the driving model of the CAV, and the ACC module was modified to implement our CACC system. However, the communication system of a PreScan is not as sophisticated as a network simulator. Therefore, we assume that the V2V communication delay has a normal distribution because limitations exist in the implementation of realistic V2V channel characteristics and V2V network protocols. The PDR is modeled with a Bernoulli distribution; however, the probability of loss is assumed to be very low because we consider a decentralized topology where each CAV communicates with the PV and FV.

In the simulation scenario, each CAV is equipped with two RADAR sensors and V2V communication devices that comply with the IEEE 802.11p/WAVE standard [36]. There are eight CAVs in the scenario, and they have a homogeneous type of vehicles. The leader vehicle drives along a set straight path and a uniform speed profile, and the rest of the vehicle drives through the implemented control system. The simulation starts with a platoon composed of seven vehicles in the first lane and a JV in the second lane, which drives right next to the second vehicle of the platoon. 

**Figure 7:**
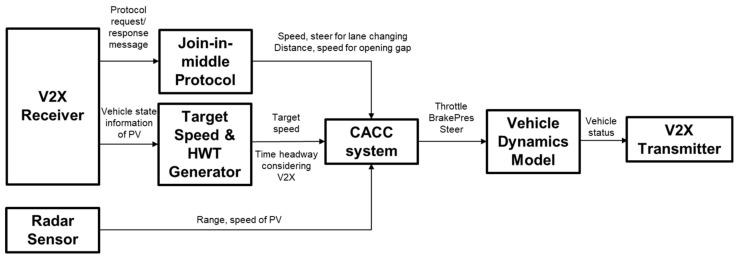
Platooning system model implemented in simulator PreScan.

Figure 7 shows the function blocks used in the simulation and the data flow. The parameters used in the simulation scenario are presented in Table 2.

### 4.2. Simulation Results

**Figure 8:**
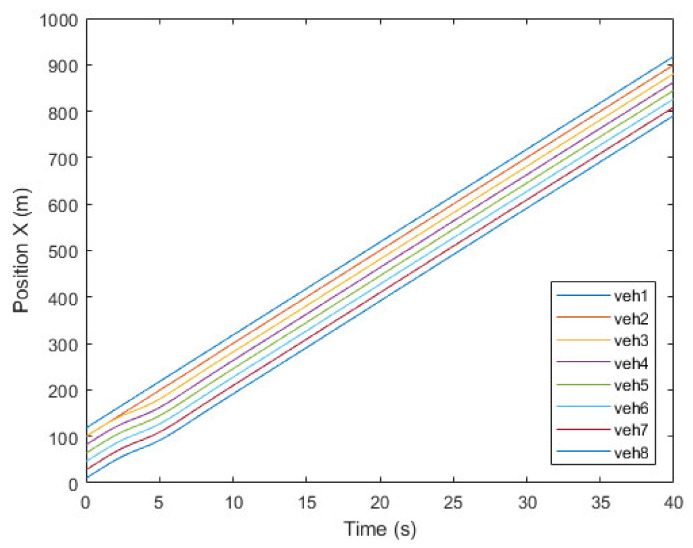
Position of vehicles.

**Figure 9:**
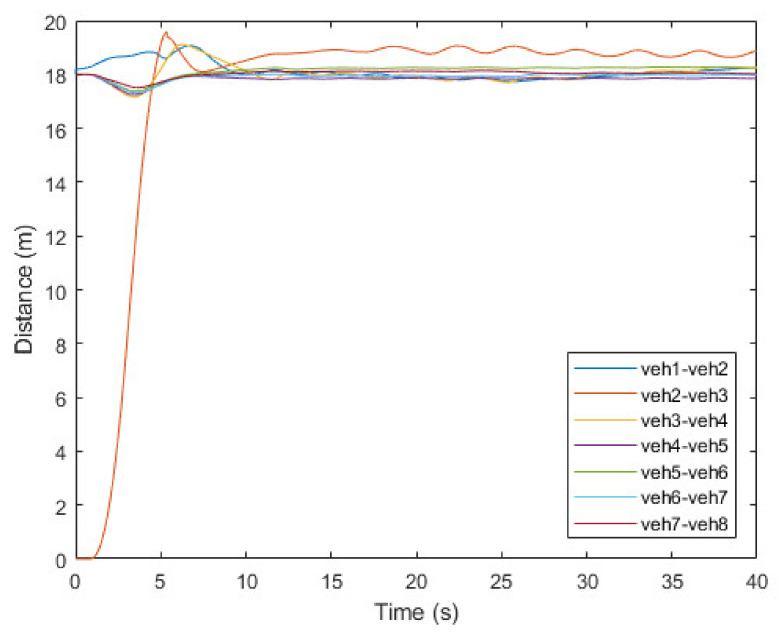
Inter vehicle distance of vehicles.

**Figure 10:**
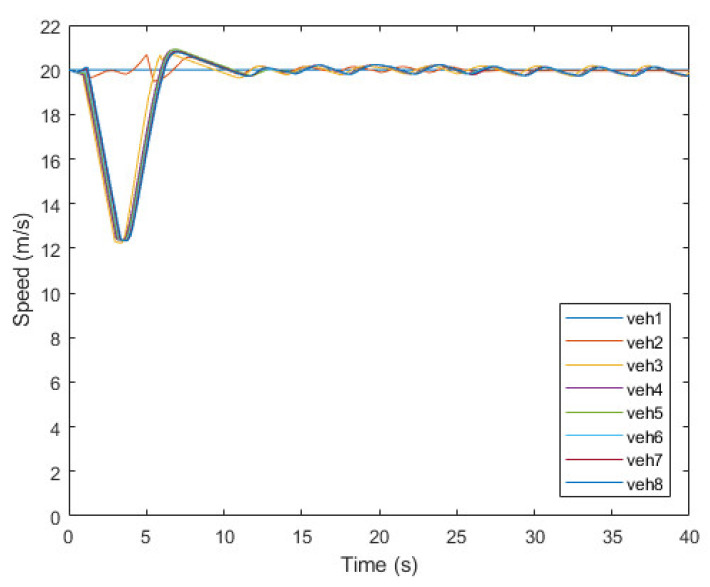
Speed of vehicles.

Figure 8, Figure 9 and Figure 10 show the simulation results of the platoon join-in-middle maneuver. Figure 8 shows the x-position of the vehicles. veh2 represents the JV and is in the same x-position as veh3 at the start. When veh2 transmits a join request message to veh1 and veh3 in 0.5 s, the join-in-middle maneuver starts. After receiving a join response message in 0.64 s, veh2 enters the preparation phase. In the preparation phase, veh2 generates a join-in-middle maneuver trajectory and transmits an open gap message to veh3. After receiving an open gap message in 0.81 s, veh3 changes the CACC system setting and starts decelerating in 0.91 s. veh3 decelerates for 2.4 s to open the gap and accelerates for 2.6 s to restore the existing platoon speed. As veh3 decelerates and accelerates, the speed of veh4–8 changes sequentially to maintain a safe distance. When the opening gap phase ends in 3.14 s, veh2 starts changing lanes to join in the platoon. After finishing the join-in-middle maneuver in 6.04 s, all vehicles drive at a similar speed.

Figure 9 shows the inter-vehicle distance based on the x-position of each vehicle. Platoon member vehicles were initially driven with an inter-vehicle distance of 18 m. The inter-vehicle distance of vehicles after veh3 is slightly reduced when the join maneuver starts and veh3 starts decelerating in 0.91 s, and veh3–8 try to restore the existing safety distance through CACC system. The result shows that the vehicle farther away from veh2 is less affected by the join-in-middle maneuver. veh4, which was directly affected by the deceleration of veh3, has an error of up to 5% compared to the reference intra-platoon distance; however, it does not affect the string stability as the distance is restored after 10 s. Although the distance between veh2 and veh3 has small fluctuation owing to lane-changing of veh2, it also does not significantly affect the safety and string stability of the platoon as a deviation of the distance is maintained within small range. Finally, the platoon recovers its string stability within 20 s after finishing the join-in-middle maneuver.

Figure 10 shows the change in speed of each vehicle. veh3 decelerates to 12.3 m/s to open the gap, and the variance is propagated to the following vehicles. After reaching its lowest speed, it immediately attempts to accelerate; however, it is delayed for 0.2 s owing to the influence of the control system. After reaching the platoon speed of 20 m/s, veh3 attempts to maintain the safety distance through PID control. Owing to the change in speed of veh3, the speed of the FVs of veh3 has small fluctuation to keep safety distance; however, it is due to limitation of PID control, and the deviation of the speed is very small, so it does not affect string stability.

Through simulation, we verified that our platoon join-in-middle maneuver does not significantly affect the string stability and operates safely in a short time. This results from direct communication with adjacent vehicles, reliable protocol procedures, and an elaborate trajectory design that considers communication delays.

## 5. Conclusions

This paper presents a decentralized platooning system for CAVs and the platoon join-in-middle maneuver protocol. The platooning system of CAVs used V2V communication to determine the target speed and safety distance and to perform platoon maneuvers. V2V communication delays and packet losses can affect the string stability of platoons. In this study, we calculated the variable time headway of the CACC system and generated a join-in-middle trajectory by considering the communication delay and packet loss. The variable time headway helps maintain a safe intra-platoon distance by reflecting the congestion of the V2V communication channel. Our join-in-middle protocol considers communication delays, retransmissions, and the opening gap delay in the FV to generate a join-in-middle trajectory; thus, the maneuver is performed quickly without affecting the string stability. The simulator PreScan was used to validate our platooning system, including V2V communication, longitude control, and lateral control, and the results showed a time of 5.5 s to finish the join-in-maneuver and a time of 25 s to restore string stability with a low-speed variance.

Several studies need to be conducted to improve our system for application to various real environments. First, research into heterogeneous vehicle platooning systems, such as those involving safe distance strategies considering various types of vehicle dynamics, is required. Second, V2V communication is affected by the environment, such as the congestion of vehicles and obstacles. Therefore, it is necessary to apply the V2V communication channel model and channel access model considering the surrounding environment in the development of a platooning system for CAVs.

## Figures and Tables

**Table 1:** Comparison of join-in-middle maneuver protocols.

Study	Network Topology	Communication Delay	Packet Loss	Spacing Strategy	Trajectory Planning
Liu et al. [31]	Centralized	Constant	Not considered	CTH	Using lateral control for lane changing
Segata et al. [32]	Centralized	Variable	Using ACK message	Constant spacing	Not considered
Renzler et al. [13]	Decentralized	Variable	Not handled	VTH	Not considered
This paper	Decentralized	Variable	Using retransmission time out	VTH	Using lateral control for lane changing

**Table 2:** Parameters used in the simulation scenario.

Parameter	Value
Simulation time	40 s
Number of CAVs	8
Length of road	1000 m
Number of lanes	2
Width of lane $y_{e}$	3.5
Initial velocity	20 m/s
$A_{cf}$	0.3 g m/$s^{2}$
$D_{cf}$	−0.35 g m/$s^{2}$
$a_{y}$	2.62 m/$s^{2}$
Width of CAV	2 m
Length of CAV	4.56 m
Mean of normal distribution	50
Standard deviation	10
Packet delivery rate	0.99
Sampling rate	10
Look up points in pure pursuit	8
$C_{x}$	2.51
$t_{processing,FV}$	50 ms
$t_{processing,JV}$	50 ms
A	0.125
β	0.25
$t_{default}$	0.5 s
$D_{standstill}$	3 m
